# Synergistic effect of renalase and chronic kidney disease on endothelin-1 in patients with coronary artery disease ‒ a cross-sectional study

**DOI:** 10.1038/s41598-018-25763-4

**Published:** 2018-05-09

**Authors:** Yu-Hsuan Li, Wayne Huey-Herng Sheu, Wen-Jane Lee, Jun -Sing Wang, Chia-Po Fu, Kae-Woei Liang, I-Te Lee

**Affiliations:** 1 Division of Endocrinology and Metabolism, Department of Internal Medicine, Taichung Veterans General Hospital, Taichung City, Taiwan; 2 School of Medicine, National Yang-Ming University, Taipei City, Taiwan; 3 Department of Medical Research, Taichung Veterans General Hospital, Taichung City, Taiwan; 4 Cardiovascular Center, Taichung Veterans General Hospital, Taichung City, Taiwan; 5 School of Medicine, Chung Shan Medical University, Taichung City, Taiwan

## Abstract

Endothelin-1 (ET-1) is associated with endothelial dysfunction and vasoconstriction. Increased circulating ET-1 levels are associated with long-term cardiovascular mortality. Renalase, released from kidney, metabolizes catecholamines and regulates blood pressure. An increase in circulating renalase levels has been reported in patients with chronic kidney disease (CKD) and is associated with coronary artery disease (CAD). We hypothesized the existence of a synergistic effect of serum renalase levels and CKD on ET-1 levels in patients with CAD. We evaluated 342 non-diabetic patients with established CAD. ET-1 and renalase levels were measured in all patients after an overnight fast. Patients with CKD had higher ET-1 (1.95 ± 0.77 vs. 1.62 ± 0.76 pg/ml, P < 0.001) and renalase levels (46.8 ± 17.1 vs. 33.9 ± 9.9 ng/ml, P < 0.001) than patients without CKD. Patients with both CKD and high renalase levels (>the median of 36.2 ng/ml) exhibited the highest serum ET-1 (P value for the trend <0.001). According to multivariate linear regression analysis, the combination of high serum renalase levels with CKD was a significant risk factor for increased serum ET-1 levels (regression coefficient = 0.297, 95% confidence interval = 0.063‒0.531, P = 0.013). In conclusion, our data suggest a synergistic effect of high serum renalase levels and CKD on increases in ET-1 levels in patients with established CAD.

## Introduction

Cardiovascular disease (CVD) is the leading cause of death in patients with chronic kidney disease (CKD)^1^. The progression of CVD and CKD are tightly linked, not only by sharing common risk factors but also by multi-system crosstalk between the kidney and the heart^2,3^. Despite enormous efforts in management of CVD risk factors, CVD prevalence remains high in patients with CKD^4^. The activation of the renin–angiotensin–aldosterone system (RAAS) system, overstimulated sympathetic activity, endothelial dysfunction, and neurohormonal imbalances together play important roles in the pathogenesis of CVD and CKD^5,6^. With the widespread use of RAAS blockers, the mortality risk has been reduced. Nevertheless, many patients progress to end-stage renal disease (ESRD) and die of CVD^7^. This suggests that RAAS blockade alone is not sufficient to prevent complications of CKD. There is an urgent need to extend the knowledge of other mechanisms and identify potential therapeutic targets.

Endothelin-1 (ET-1) is a potent vasoconstrictor^8^. It is produced by the endothelium and acts on vascular smooth muscle^9^. Elevated circulating ET-1 levels increase vascular tone and inflammation, contributing to the development of atherosclerosis and hypertension^10^. ET- 1 is a predictor of CVD and mortality^11–13^. Furthermore, ET-1 acts on the renal collecting duct and vasculature, and takes part in the deterioration of renal function^14^.

Renalase, a flavin adenine dinucleotide-dependent amine oxidase produced by the kidney, metabolizes catecholamines and may be a therapeutic target for the management of the overstimulated sympathetic system^15^. Renalase was reported to attenuate renal fibrosis in a rat model of ureteral obstruction^16^. However, circulating renalase levels were reported to be inversely correlated with the estimated glomerular filtration rate (eGFR)^17,18^, and might predict renal-function decline in recipients of renal transplants^19^. Renalase may also predict disease activity in patients with lupus nephritis^20^. Recently, it has been hypothesized that circulating renalase may be a risk factor for CVD^21,22^. Since the mechanistic bridge between CKD and CAD has yet to be elucidated, we investigated the effect of renalase and CKD on ET-1 levels in patients with established CAD.

## Materials and Methods

### Study design and subjects

This cross-sectional study was conducted in the outpatient section of Taichung Veterans General Hospital between May 2009 and December 2016. The inclusion criteria were: (1) age >20 years, and (2) a history of myocardial infarction, coronary artery lesions with significant lumen narrowing ≥50%, or coronary revascularization. The exclusion criteria were: (1) unstable ischemic heart disease, (2) a history of diabetes or treatment with anti-diabetic drugs, (3) ongoing treatment for psychological disorders, (4) presence of acute infectious diseases, (5) severe systemic diseases such as malignancy or immune disorder, (6) end-stage renal disease treated by dialysis, and (7) pregnancy. The study complied with the Declaration of Helsinki and was approved by the Institutional Review Board of Taichung Veterans General Hospital. Written informed consent was obtained from all participants before study procedures were performed. All methods were performed in accordance with the relevant guidelines and regulations.

### Study procedures

Blood pressure was measured at right brachial artery, and the mean of two separate measurements with intervals of 1 minute was recorded after subjects had sat and rested for 5 minutes (DINAMAPTM^®^, DPC3000M-EN, GE Healthcare, WI, USA). Waist circumference was measured at the level of middle distance between the last rib margin and the upper ilial border after expiration, while the participant breathed quietly and smoothly (kp-1508, King Life, Taipei, Taiwan). Blood samples were collected in the morning after an overnight fast. Plasma was prepared to measure glucose concentrations, and serum was prepared to measure lipid, C-reactive protein (CRP), creatinine, renalase and ET-1 levels. A spot urine sample was collected to measure urine albumin and creatinine levels. Plasma samples were prepared using EDTA as an anticoagulant and were removed for glucose measurement immediately after centrifugation within 30 minutes of collection. Serum samples were prepared using a serum separator tube for approximately 30 minutes at room temperature before centrifugation. Serum samples were stored at −80 °C and were first thawed for these assays.

### Definitions of anthropometric data and lab results

Obesity was defined as a body mass index (BMI) >27 kg/m^2^ according to standards for the Taiwanese population^23^. According to the criteria for components of metabolic syndrome from the National Cholesterol Education Program (NCEP)^24^, central obesity was defined as a waist circumference >90 cm in men or >80 cm in women. Hypertension was defined as a blood pressure ≥130/85 mm Hg or current use of antihypertensive medications. Hypertriglyceridemia was defined as serum triglyceride levels ≥150 mg/dl (1.7 mmol/L). Low high-density lipoprotein (HDL) cholesterol was defined as a serum HDL cholesterol concentration <40 mg/dl (1.0 mmol/L) in men and <50 mg/dl (1.3 mmol/L) in women. Impaired fasting glucose was defined as a fasting glucose concentration ≥100 mg/dl (5.6 mmol/L). Metabolic syndrome was diagnosed if three or more of the above five components were present. Based on the Modification of Diet in Renal Disease (MDRD) equation^25^, the eGFR was calculated as 186 × [serum creatinine concentration (mg/dl)]^−1.154^ × [age (year)]^−0.203^ (×0.742, if female), and CKD was defined as an eGFR <60 ml/min/1.73 m^2^. Urine albumin creatinine ratio (ACR) was determined by the ratio of urine albumin (mg) to urine creatinine (g)^26^.

### Biochemical analyses

Glucose levels were determined using the oxidase-peroxidase method (Wako Diagnostics, Tokyo, Japan). Creatinine and lipid concentrations were determined using the commercial kits (Beckman Coulter, Fullerton, USA). CRP levels were determined using an immunochemical assay employing purified duck IgY (∆Fc) antibodies (Good Biotech Corp., Taichung, Taiwan). Urinary albumin levels were determined using the polyethylene glycol-enhanced immunoturbidimetric method (Advia 1800, Siemens, New York, USA). Serum human ET-1 levels were determined using an enzyme-linked immunosorbent assay (ELISA) (R&D Systems, Minneapolis, USA). The intra-assay coefficient of variation (CV) for the ET-1 measurement was 4.0%, and the inter-assay CV was 7.6%. The analytical sensitivity for the ET-1 measurement was 0.087 pg/ml. Serum renalase concentrations were determined by an ELISA (Wuhan USCN Business Co., Wuhan, China). The analytical sensitivity of the renalase measurement was 1.31 ng/ml. Precision within an assay was assessed by measuring three samples with low, middle and high levels of renalase, respectively, in 20 replicates on one plate. The intra-assay CV for renalase was less than 10.0%. Precision between assays was assessed by measuring three samples with low, middle and high levels of renalase, respectively, at identical positions of eight different plates. The inter-assay CV for renalase was less than 12.0% based on these six replicates. Serum samples were stored for less than five years before analysis. We evaluated the reproducibility of the renalase concentration between two measurements in a group of 40 samples collected before January 2014. The reproducibility of renalase measurements showed a high linear correlation, with a correlation coefficient (*r*) of 0.963 (P < 0.001) and a bias of −0.39 ± 4.82 between repeated measurement based on the results of the Bland-Altman analysis.

### Statistical analysis

All continuous data are presented as means ± standard deviation (SD), and categorical data are presented as numbers (percentages). Statistical analyses were conducted using the independent sample *t*-test to detect statistically significant differences in continuous variables between two groups, and using one-way analysis of variance (ANOVA) for more than two groups. The chi-squared test was used to detect differences in categorical variables. A test for trends in serum ET-1 concentrations was performed across the four groups categorized by CKD and the median serum renalase level. Correlations between two variables were assessed by calculating Pearson’s correlation coefficients. Linear regression analyses were conducted to identify factors associated with serum ET-1 levels. Statistical analyses were performed with SPSS version 22.0 software (IBM Corp., Armonk, NY, USA).

## Results

### Characteristics of patients with and without CKD

Of the total of 342 patients with established CAD, 85 with an eGFR <60 ml/min/1.73 m^2^ were allocated to the CKD(+) group, and 257 with an eGFR ≥60 ml/min/1.73 m^2^ were allocated to the CKD(−) group. The characteristics of the patients in these two groups are displayed in Table 1. Patients in the CKD(+) group were older than patients in the CKD(−) group (69 ± 10 vs. 60 ± 11 years, P < 0.001). Patients in the CKD(+) group had higher systolic blood pressures than those in the CKD(−) group (132 ± 19 vs. 126 ± 18 mmHg, P = 0.010). Patients in the CKD(+) group had higher CRP levels than those in the CKD(−) group (3.2 ± 2.7 vs. 2.3 ± 2.3 mg/L, P = 0.004). We detected higher urine ACR and lower eGFR in patients in the CKD(+) group compared with that of patients in the CKD(−) group (96 ± 244, vs. 33 ± 101 for ACR, and 48 ± 11 vs. 82 ± 16 ml/min/1.73 m^2^ for eGFR; both P values < 0.001). We detected higher serum ET-1 and renalase levels in the CKD(+) group than in the CKD(−) group (1.95 ± 0.77 vs. 1.62 ± 0.76 pg/ml, P < 0.001 for ET-1; 46.8 ± 17.1 vs. 33.9 ± 9.9 ng/ml, P < 0.001 for renalase).

**Table 1:** Clinical characteristics of patients stratified by an estimated glomerular filtration rate (eGFR) of 60 ml/min/1.73 m^2^.

	All (N = 342)	eGFR <60 (N = 85)	eGFR ≥60 (N = 257)	P^#^
**Demographic characteristics**				
Age (year)	62 ± 11	69 ± 10	60 ± 11	<0.001
Male, n (%)	304 (88.9%)	74 (87.1%)	230 (89.5%)	0.674
Current smoker, n (%)	211 (61.7%)	46 (54.1%)	165 (64.2%)	0.126
Hypertension, n (%)	325 (95.0%)	78 (91.8%)	247 (96.1%)	0.190
**Anthropometric data**				
BMI (kg/m^2^)	26.2 ± 3.7	25.8 ± 3.5	26.3 ± 3.8	0.264
Waist circumference (cm)	91.5 ± 9.1	91.3 ± 8.8	91.5 ± 9.2	0.847
Systolic blood pressure (mmHg)	128 ± 19	132 ± 19	126 ± 18	0.010
Diastolic blood pressure (mmHg)	74 ± 10	73 ± 10	75 ± 11	0.324
**Glucose/lipoprotein/CRP**				
Fasting glucose (mmol/L)	5.4 ± 0.8	5.3 ± 1.0	5.4 ± 0.7	0.781
Triglycerides (mmol/L)	1.5 ± 0.8	1.7 ± 1.1	1.4 ± 0.7	0.034
Total cholesterol (mmol/L)	4.2 ± 1.0	4.3 ± 1.0	4.2 ± 1.0	0.825
HDL cholesterol (mmol/L)	1.2 ± 0.3	1.2 ± 0.3	1.2 ± 0.3	0.554
LDL cholesterol (mmol/L)	2.4 ± 0.9	2.4 ± 0.9	2.4 ± 0.9	0.834
CRP (mg/L)*	2.5 ± 2.4	3.2 ± 2.7	2.3 ± 2.3	0.004
**Renal function**				
Albumin to creatinine ratio (mg/g)*	48 ± 152	96 ± 244	33 ± 101	<0.001
eGFR (ml/min/1.73 m^2^)	73 ± 21	48 ± 11	82 ± 16	<0.001
**Number of vessels displaying significant narrowing on coronary angiography**				0.968
No vessel but MI history, n (%)	38 (11.1%)	10 (11.8%)	28 (10.9%)	
One vessel, n (%)	127 (37.1%)	33 (38.8%)	94 (36.6%)	
Two vessels, n (%)	102 (29.8%)	24 (28.2%)	78 (30.4%)	
Three vessels, n (%)	75 (21.9%)	18 (21.2%)	57 (22.2%)	
**Cardiorenal factors**				
Endothelin-1 (pg/ml)	1.70 ± 0.77	1.95 ± 0.77	1.62 ± 0.76	<0.001
Renalase (ng/ml)	37.1 ± 13.3	46.8 ± 17.1	33.9 ± 9.9	<0.001
**Current medications**				
Antihypertensive agents, n (%)	317 (92.7%)	76 (89.4%)	241 (93.8%)	0.272
ACE inhibitor or ARB, n (%)	229 (67.0%)	57 (67.1%)	172 (66.9%)	0.999
α-blocker, n (%)	22 (6.4%)	6 (7.1%)	16 (6.2%)	0.987
β-blocker, n (%)	102 (29.8%)	31 (36.5%)	71 (27.6%)	0.159
Calcium channel blocker, n (%)	173 (50.6%)	42 (49.4%)	131 (51.0%)	0.901
Diuretics, n (%)	60 (17.5%)	18 (21.2%)	42 (16.3%)	0.395
Antiplatelet, n (%)	333 (97.4%)	81 (95.3%)	252 (98.1%)	0.323
Statins, n (%)	257 (75.1%)	59 (69.4%)	198 (77.0%)	0.205

ACE = angiotensin-converting enzyme, ARB = angiotensin II receptor blocker, BMI = body mass index, CRP = C-reactive protein, eGFR = estimated glomerular filtration rate, HDL = high-density lipoprotein, LDL = low-density lipoprotein, MI = myocardial infarction.

*Values were logarithm-transformed (log) in the analyses due to a skewed distribution.

^#^P values for differences between the two groups.

### Risk factors associated with elevated serum ET-1 levels

Using the median serum renalase level (36.2 ng/ml) as a cutoff, a higher mean serum ET-1 level was detected in patients with serum renalase levels ≥36.2 ng/ml than in patients with renalase levels <36.2 ng/ml (1.81 ± 0.86 vs. 1.59 ± 0.66 pg/ml, P = 0.009). A higher mean serum ET-1 level was observed in patients aged ≥60 years than in those aged <60 years (1.77 ± 0.68 vs. 1.60 ± 0.87 pg/ml, P = 0.044). A higher mean serum ET-1 level was recorded in patients with CRP levels ≥2 mg/L than in those with CRP levels <2 mg/L (1.94 ± 0.88 vs. 1.50 ± 0.60 pg/ml, P < 0.001). Mean serum ET-1 levels were higher in patients with urine ACR ≥30 mg/g than in patients with normoalbuminuria (1.98 ± 1.09 vs. 1.63 ± 0.65 pg/ml, P < 0.001), and were higher in patients using diuretics than in those not using diuretics (1.92 ± 1.08 vs. 1.65 ± 0.68 pg/ml, P = 0.013, Table 2).

**Table 2:** Serum endothelin-1 levels in patients stratified by associated risk factors.

	N(%)	Endothelin-1 (pg/ml) Mean ± SD	P
Age (year)			0.044
<60	146 (42.7%)	1.60 ± 0.87	
≥60	196 (57.3%)	1.77 ± 0.68	
Male			0.219
No	38 (11.1%)	1.84 ± 0.68	
Yes	304 (88.9%)	1.68 ± 0.78	
Current smoker			0.471
No	131 (38.3%)	1.66 ± 0.65	
Yes	211 (61.7%)	1.72 ± 0.84	
BMI (kg/m^2^)			0.407
<24	100 (29.2%)	1.79 ± 0.92	
24‒26.9	118 (34.5%)	1.68 ± 0.71	
≥27	124 (36.3%)	1.65 ± 0.70	
MetS			0.455
No	171 (50.0%)	1.67 ± 0.67	
Yes	171 (50.0%)	1.73 ± 0.86	
Waist circumference >90 cm in men and >80 cm in women			0.679
No	146 (42.7%)	1.72 ± 0.90	
Yes	196 (57.3%)	1.68 ± 0.66	
BP ≥130/85 mmHg or using anti-hypertensive drugs			0.329
No	17 (5.0%)	1.52 ± 0.75	
Yes	325 (95.0%)	1.71 ± 0.77	
Fasting glucose ≥5.6 mmol/L			0.508
No	243 (71.1%)	1.68 ± 0.67	
Yes	99 (28.9%)	1.74 ± 0.95	
Triglycerides >1.7 mmol/L			0.564
No	238 (69.6%)	1.72 ± 0.78	
Yes	104 (30.4%)	1.66 ± 0.75	
HDL cholesterol <1.0 mmol/L in men and <1.3 mmol/L in women			0.171
No	225 (65.8%)	1.66 ± 0.65	
Yes	117 (34.2%)	1.78 ± 0.96	
Total cholesterol ≥4.1 mmol/L			0.751
No	148 (43.3%)	1.71 ± 0.79	
Yes	181 (52.9%)	1.68 ± 0.74	
LDL cholesterol ≥1.8 mmol/L			0.221
No	63 (18.4%)	1.80 ± 0.59	
Yes	227 (66.4%)	1.66 ± 0.83	
CRP ≥2 mg/L			<0.001
No	189 (55.3%)	1.50 ± 0.60	
Yes	153 (44.7%)	1.94 ± 0.88	
Albumin to creatinine ratio ≥30 mg/g			<0.001
No	270 (78.9%)	1.63 ± 0.65	
Yes	72 (21.1%)	1.98 ± 1.09	
Renalase ≥36.2 ng/ml*			0.009
No	171 (50.0%)	1.59 ± 0.66	
Yes	171 (50.0%)	1.81 ± 0.86	
Number of vessels displaying significant narrowing on coronary angiography			0.885
0 vessel	38 (11.1%)	1.70 ± 0.67	
1 vessel	127 (37.1%)	1.70 ± 0.89	
2 vessels	102 (29.8%)	1.74 ± 0.72	
3 vessels	75 (21.9%)	1.65 ± 0.67	
Current use of ACE inhibitor or ARB			0.163
No	113 (33.0%)	1.62 ± 0.70	
Yes	229 (67.0%)	1.74 ± 0.80	
Current use of α-blocker			0.179
No	320 (93.6%)	1.68 ± 0.78	
Yes	22 (6.4%)	1.91 ± 0.70	
Current use of β-blocker			0.701
No	240 (70.2%)	1.71 ± 0.82	
Yes	102 (29.8%)	1.67 ± 0.66	
Current use of calcium channel blocker			0.655
No	169 (49.4%)	1.72 ± 0.90	
Yes	173 (50.6%)	1.68 ± 0.62	
Current use of diuretics			0.013
No	282 (82.5%)	1.65 ± 0.68	
Yes	60 (17.5%)	1.92 ± 1.08	
Current use of statins			0.315
No	85 (24.9%)	1.77 ± 0.83	
Yes	257 (75.1%)	1.68 ± 0.75	

ACE = angiotensin-converting enzyme, ARB = angiotensin II receptor blocker, BMI = body mass index, CI = confidence interval, CRP = C-reactive protein, HDL = high-density lipoprotein, LDL = low-density lipoprotein, MetS = metabolic syndrome.

*Cutoff value based on the median.

### Synergistic effect of renalase and CKD on serum ET-1 levels

**Figure 1:**
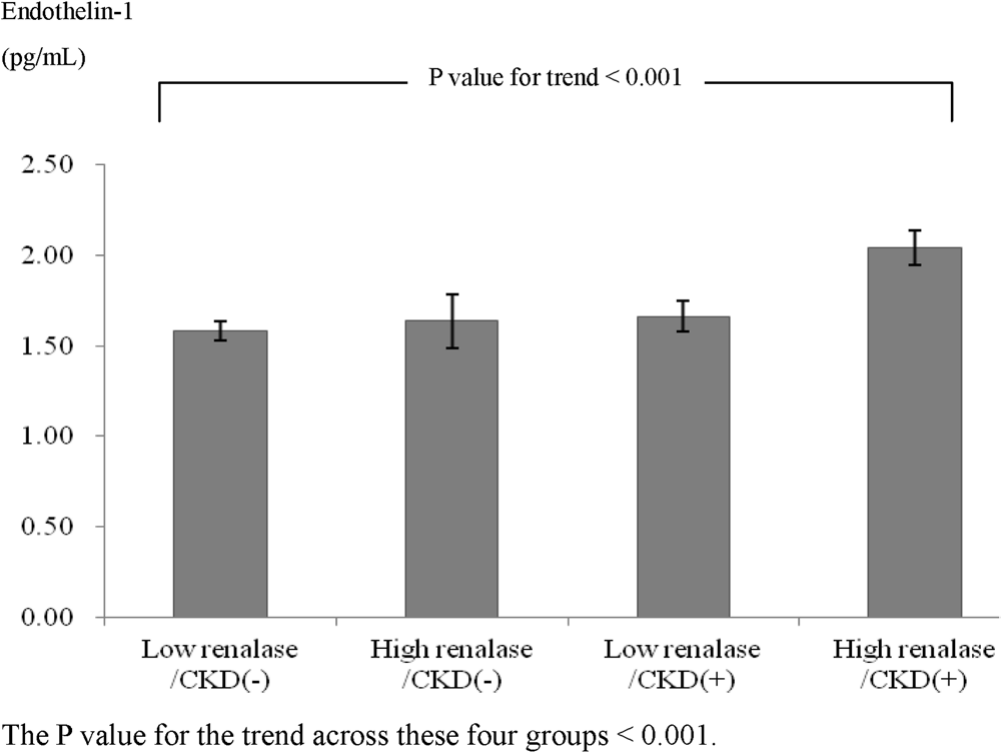
Serum endothelin-1 concentrations and standard error bars in different groups stratified by serum renalase levels and the presence of chronic kidney disease (CKD).

As shown in Table 3, serum ET-1 levels were positively correlated with serum renalase levels (*r* = 0.136, P = 0.012) and inversely correlated with the eGFR (*r* = −0.191, P < 0.001). Furthermore, a significant correlation between serum renalase levels and eGFR was observed (*r* = −0.419, P < 0.001). Based on the present or absence of CKD and the median serum renalase level, patients were divided into four groups: low renalase/CKD(−), low renalase/CKD(+), high renalase/CKD(−), and high renalase/CKD(+). Serum ET-1 levels were 1.59 ± 0.66, 1.64 ± 0.66, 1.66 ± 0.87, and 2.04 ± 0.78 pg/ml in these four groups, respectively. Figure 1 shows a significant positive trend for serum ET-1 levels from the low renalase/CKD(−) group to the high renalase/CKD(+) group (P value for the trend <0.001).

**Table 3:** Pearson’s correlation coefficients (*r*) for the correlations between serum endothelin-1 (ET-1), renalase and estimated glomerular filtration rate (eGFR).

	ET-1	Renalase	eGFR
ET-1			
Renalase	0.136 (P = 0.012)		
eGFR	−0.191 (P < 0.001)	−0.419 (P < 0.001)	

eGFR = estimated glomerular filtration rate, ET-1 = endothelin-1.

Serum ET-1 levels also significantly correlated with age, diastolic blood pressure, fasting glucose, urine ACR, and using diuretics according to the univariate regression analysis (Table 4). Based on the results of the multivariate regression analysis, high renalase/CKD(+) was a dependent risk factor for higher serum ET-1 levels after adjustment for gender and other risk factors identified in the univariate regression analysis (regression coefficient = 0.297, 95% confidence interval = 0.063‒0.531, P = 0.013).

**Table 4:** Effects of risk factors on serum endothelin-1 levels.

	Univariate model			Multivariate model*					
	Crude			Model 1			Model 2		
	B	95%CI	P	B	95%CI	P	B	95%CI	P
Low renalase/CKD(−)	reference			reference			reference		
High renalase/CKD(−)	0.053	(−0.301, 0.407)	0.770	0.051	(−0.305, 0.408)	0.777	0.023	(−0.320, 0.366)	0.895
Low renalase/CKD(+)	0.077	(−0.111, 0.266)	0.421	0.084	(−0.108, 0.276)	0.392	0.079	(−0.106, 0.263)	0.402
High renalase/CKD(+)	0.460	(0.239, 0.680)	<0.001	0.418	(0.181, 0.656)	<0.001	0.297	(0.063, 0.531)	0.013
Age (every 1 year)	0.009	(0.002, 0.016)	0.013	0.004	(−0.004, 0.011)	0.368	0.002	(−0.006, 0.010)	0.634
Male	−0.164	(−0.425, 0.097)	0.219	−0.130	(−0.393, 0.134)	0.334	−0.148	(−0.401, 0.106)	0.252
Current smoker (yes/no)	0.062	(−0.107, 0.231)	0.471						
BMI (every 1 kg/m^2^)	−0.008	(−0.030, 0.014)	0.487						
Systolic BP (every 1 mmHg)	0.004	(−0.002, 0.010)	0.154						
Diastolic BP (every 1 mmHg)	0.008	(0.000, 0.016)	0.041				−0.002	(−0.010, 0.006)	0.577
Fasting glucose (every 1 mmol/L)	0.125	(0.020, 0.229)	0.020				0.091	(−0.009, 0.192)	0.075
Triglycerides (every 1 mmol/L)	0.012	(−0.088, 0.111)	0.818						
Total cholesterol (every 1 mmol/L)	−0.081	(−0.161, 0.000)	0.051						
HDL cholesterol (every 1 mmol/L)	−0.274	(−0.575, 0.027)	0.074						
LDL cholesterol (every 1 mmol/L)	−0.081	(−0.172, 0.011)	0.086						
MetS (yes/no)	0.062	(−0.102, 0.227)	0.455						
Urine ACR ≥30 (yes/no)	0.283	(0.159, 0.407)	<0.001				0.183	(−0.017, 0.382)	0.073
CRP ≥2 mg/L (yes/no)	0.438	(0.279, 0.596)	<0.001				0.345	(0.184, 0.506)	<0.001
Current use of ACE inhibitor or ARB	0.124	(−0.050, 0.298)	0.163						
Current use of diuretics	0.272	(0.058, 0.486)	0.013				0.190	(−0.015, 0.395)	0.070
Current use of statins	−0.097	(−0.287, 0.093)	0.315						

B = linear regression coefficient, which represents the mean change in serum endothelin-1 levels for per unit increase in the levels of the associated risk factor (for continuous variables) or compared to the reference group (for categorical variables).

ACE = angiotensin-converting enzyme, ARB = angiotensin II receptor blocker, BMI = body mass index, CI = confidence interval, CKD = chronic kidney disease, HDL = high-density lipoprotein, LDL = low-density lipoprotein, MetS = metabolic syndrome.

^*^Multivariate linear regression analysis.

## Discussion

We found that either elevated serum renalase or low eGFR was associated with elevated serum ET-1 levels. The presence of both high serum renalase and CKD showed the highest serum ET-1 levels among patients with established CAD. To our knowledge, this is the first report demonstrating serum ET-1 levels associated with both serum renalase and CKD. Our data suggest a synergistic effect of renalase and CKD on increasing serum ET-1 levels in non-diabetic patients with CAD.

The pathogenesis of CVD in patients with CKD is complex^1,27^. The main culprits include an overstimulated RAAS, sympathetic activation, chronic inflammation, and endothelial dysfunction. All these factors interact with one another in a vicious cycle^6,28^. Identification of the mediator between kidney and heart is important for therapeutic targeting of cardiorenal syndrome^29^. Evidence suggested renalase secreted from kidney is associated with CVD^21^. However, the mechanism by which circulating renalase affects CKD is unknown. There are inconsistent findings across studies^22^. In a mouse knockout model, renalase deficiency was related to more extensive ischemic myocardial damage than that in wild-type mice^30^. In a rat model of subtotal nephrectomy, systemic renalase supplementation prevented cardiomyocyte fibrosis and ameliorated cardiac remodeling^31^. Despite renal renalase expression increased within one week and decreased after second week post acute myocardial infarction (MI), circulating renalase levels continued to be elevated four weeks post-MI in a rat model^32^. In our study, patients were enrolled after the stabilization of their cardiovascular conditions. In line with our findings, Baek *et al*. reported that high serum renalase levels predicted all-cause mortality in a Korean study^33^. One strength of our study is that we considered the confounding effect of CKD on serum renalase concentrations.

The association between CKD and ET-1 has been well documented^34^. Excessive ET-l production may drive CKD progression by causing acute ischemic renal injury, renal fibrosis, or podocyte dysfunction^35^. Blockade of ET-1 receptors may prevent renal inflammation and fibrosis^7^. Elevated circulating ET-1 released from arteries was found following nephrectomy in hypertensive rats^36^. Similarly, Ruschitzka *et al*. found that circulating ET-1 and renal ET-1 increased with vascular endothelial dysfunction following acute renal failure in a rat model^37^. Therefore, renal damage might induce systemic overexpression of ET-1, and might participate in cardiovascular pathogenesis in CKD. Przybylowski *et al*.^38^ reported that serum renalase might increase in heart transplant recipients, and this increase in serum renalase might be caused by a decrease in renal function. Since a synergistic effect of renalase and CKD on serum ET-1 in our study, it is reasonable to speculate that increased renalase in CKD may increase circulating ET-1, which aggravates CV risk in patients with CAD.

In the present study, CRP was an independent risk factor for increased serum ET-1 levels. Consistent with the results from our study, a positive association between circulating CRP and ET-1 levels has been found in patients after ischemic stroke^39^. Dow *et al*.^40^ reported that CRP induced an increase in circulating ET-1 levels in rats with diabetes. In the study by Ramzy *et al*.^41^, ET-1 accentuated the effect of CRP on endothelial dysfunction in an *in vitro* model of endothelial cells. However, circulating CRP levels were not significantly associated with endothelial function after an intra-arterial ET-1 infusion in a human study^42^. The causal relationship between these factors requires further investigation.

In addition to CKD and serum renalase levels, use of diuretics was significantly associated with increase in serum ET-1 levels. As shown in the study by van Kraaij *et al*.^43^, circulating ET-1 levels were not significantly altered after three-month withdrawal of diuretics in a randomized, placebo-controlled, double-blinded trial. Galve *et al*.^44^ reported that fasting glucose improved, but ET-1 levels were not significantly altered after diuretic withdrawal in patients with stabilized heart failure. Long-term use of diuretics, but neither calcium channel blockers nor β blockers, increased the risk of new-onset diabetes in the Nateglinide and Valsartan in Impaired Glucose Tolerance Outcomes Research (NAVIGATOR) trial^45^. The use of thiazide diuretics as an anti-hypertensive treatment has been reported to significantly increase fasting glucose levels in a meta-analysis study^46^. High glucose levels might induce ET-1 secretion from *in vitro* aortic endothelial cells^47^. Circulating ET-1 levels were higher in patients with type 2 diabetes than in healthy controls, and ET-1 levels exhibit a positive correlation with fasting glucose levels^48^. In the present study, fasting glucose levels were associated with high serum ET-1 levels in univariate model. However, neither fasting glucose levels nor the use of diuretics was significantly associated with ET-1 levels in the multivariate regression analysis.

In the present study, the number of significantly narrowed coronary vessels was not associated with CKD, potentially because all enrolled patients were diagnosed with CAD. Consistent with our findings, a significantly lower eGFR was observed in patients with CAD than in patients without CAD, but the eGFR was not significantly different among patients with CAD presenting with different numbers of narrowed coronary vessels based on multi-detector row computed tomography^49^.

There were some limitations in our study. First, this study employed a cross-sectional design, and therefore we cannot interpret casual links. Second, we did not assess the real source responsible for the increased renalase or ET-1 release. Third, we did not analyze the cause of CKD, which is a complex disease with various pathogeneses. Finally, we did not stratify CKD stage due to the limited sample size.

In conclusion, serum renalase levels were higher in the patients with CKD than those without CKD. There was a synergistic effect of serum renalase and CKD on increases in serum ET-1 levels in patients with established CAD. However, the casual relation between ET-1 and renalase requires further investigations.
